# Non-participation in population-based disease prevention programs in general practice

**DOI:** 10.1186/1471-2458-12-856

**Published:** 2012-10-09

**Authors:** Berber Koopmans, Mark MJ Nielen, François G Schellevis, Joke C Korevaar

**Affiliations:** 1 Netherlands Institute for Health Services Research, Utrecht, The Netherlands

**Keywords:** General practice, Prevention, Vaccination, Screening, Non-participation

## Abstract

**Background:**

The number of people with a chronic disease will strongly increase in the next decades. Therefore, prevention of disease becomes increasingly important. The aim of this systematic review was to identify factors that negatively influence participation in population-based disease prevention programs in General Practice and to establish whether the program type is related to non-participation levels.

**Methods:**

We conducted a systematic review in Pubmed, EMBASE, CINAHL and PsycINFO, covering 2000 through July 6th 2012, to identify publications including information about characteristics of non-participants or reasons for non-participation in population-based disease prevention programs in General Practice.

**Results:**

A total of 24 original studies met our criteria, seven of which focused on vaccination, eleven on screening aimed at early detection of disease, and six on screening aimed at identifying high risk of a disease, targeting a variety of diseases and conditions. Lack of personal relevance of the program, younger age, higher social deprivation and former non-participation were related to actual non-participation. No differences were found in non-participation levels or factors related to non-participation between the three program types. The large variation in non-participation levels within the program types may be partly due to differences in recruitment strategies, with more active, personalized strategies resulting in higher participation levels compared to an invitation letter.

**Conclusions:**

There is still much to be gained by tailoring strategies to improve participation in those who are less likely to do so, namely younger individuals, those living in a deprived area and former non-participants. Participation may increase by applying more active recruitment strategies.

## Background

The number of people with a chronic disease will strongly increase in the next decades. For example, the worldwide prevalence of diabetes mellitus, is expected to increase by 54% from 2010 to 2030 [1]. Chronic diseases are responsible for a considerable burden to both the individual and to the healthcare system. Consequently, it is expected that the demand on health care will rise excessively, and preservation of good quality healthcare becomes unaffordable.

In order to reduce the disease burden for people and to keep healthcare affordable, a shift from treatment of an individual with a disease towards maintenance of health or postponement of disease, becomes inevitable.

General Practitioners (GPs) play an important role in prevention, since they deliver comprehensive, holistic and easily accessible care. GPs generally provide preventive care in the individual setting of a consultation, either on the indication of present risk factors and symptoms of disease or care-related, for example to reduce the risk of complications of chronic disease. Population-based prevention has a less prominent role in General Practice [2]. Population-based prevention programs initiated by GPs potentially contribute significantly to reduce the risk of developing chronic diseases or to identify treatable diseases at an early stage (disease prevention).

Current examples of population-based disease prevention programs embedded in General Practice include vaccination programs (e.g. vaccination for influenza) and screening programs (e.g. screening for cervical cancer). The purpose of vaccination is to prevent infectious diseases or to diminish the impact of infectious diseases. Population-based screening aims at early detection of a disease, thereby increasing the chance of successful treatment and diminishing the impact of the disease, or it intends to identify persons with a high risk of developing a chronic condition, whereby changes in lifestyle and/or use of medication could reduce this risk substantially. Prevention programs embedded in General Practice practices are usually applied in individuals with a potential elevated risk of disease, since this is thought to be the most (cost) effective approach [3]; only those who might benefit most are invited, whereas the number of those who undergo clinical measurements is minimized. Furthermore, in this manner workload in General Practice is limited.

The potential gains of prevention in persons with an elevated risk of disease are considerable, yet the challenges in changing health risks are numerous. Health benefits of the total population increase by increasing participation levels of high risk individuals. However, participation levels are often suboptimal and differ between groups. To increase this level, it is important to know which individuals are less likely to participate and to understand their reasons and barriers. Previous studies showed that numerous socio-demographic and behavioural factors may play a role, as well as factors on a community or organizational level [4-8]. In addition, factors, such as the nature of the target condition [9] and the program itself [6], may also play a role in the willingness to participate. Finally, the method of recruitment might influence the participation level as well [6].

The aim of this systematic review is to identify factors that negatively influence participation in population-based disease prevention programs in General Practice and to establish whether the program type is related to participation levels.

## Methods

### Data sources and searches

A systematic literature search was conducted in Pubmed, EMBASE, CINAHL and PsycINFO to identify relevant articles published between January 1^st^, 2000 and July 6th, 2012. Language was restricted to English and Dutch. The search strategy was formulated in Pubmed and adapted to the other databases (see Additional file 1: Appendix 1 for the search strategy). When necessary, equivalents for the MeSH terms were used or MeSH terms were used as free text words.

### Study selection

The first two stages of the selection for inclusion; in succession, the screening of titles, and abstracts, were performed by the first author (BK). JK also screened a random 10% of the abstracts and interrater agreement was high (kappa 0.89). All articles were screened on full text by two authors according to the criteria presented in Additional file 2: Box 1.

We aimed to select articles including information on non-participation in population-based disease prevention programs aimed at high-risk individuals in General Practice. This type of programs include an active approach of inviting a pre-defined target group to undergo screening or vaccination.

The most important inclusion criteria were:

– Programs had to be population based, and therefore not related to the individual care setting (e.g. opportunistic screening following complaints of the patient), and

– aimed at high risk individuals.

– Programs needed to be performed in General Practice.

– Studies needed to give information on either characteristics or reasons of non-participation or both.

Furthermore, to be included, articles had to describe original studies.

BK screened all articles, JK and MN screened each half of the articles. Disagreements were solved by the authors who screened the article, in case of no consensus, the third reviewer was consulted and the majority decided.

### Data extraction

For each study, the following data were abstracted:

1. general information: first author, year of publication, design, sample size and country where the study was conducted.

2. general information of the program: target disease, inclusion criteria of the target group, recruitment strategy, sending of reminders, variables used to compare participants and non-participants.

3. information on participation levels and willingness to participate.

For reasons of efficiency, only the significant differences between non-participants and participants were mentioned in the results section. In addition, results were presented in a narrative summary of characteristics that negatively influence participation, rather than in terms of effect sizes.

### Data synthesis

Studies were grouped based on the goal of the program (program type): 1) vaccination, 2) early detection of disease and 3) identification of high risk of a disease. Furthermore, to avoid duplicate presentation of the same data we grouped studies within the same population. Non-participation levels, significant characteristics of non-participants and their reasons when mentioned in both studies were only presented once, using the results of the study including the largest sample size within the same population.

### Assessment of study quality

Existing quality assessment instruments mainly focus on the quality around the primary outcome, rather than to secondary issues as is the case in our review. Therefore, we assessed quality of the studies based on the presence (or absence) of our main inclusion criterion. Studies not including characteristics or reasons of non-participants were found of insufficient quality and therefore excluded. Studies including characteristics or reasons of non-participants were found of moderate quality and received one asterisk, studies including both characteristics AND reasons of non-participants were found of higher quality and received two asterisks.

## Results

**Figure 1:**
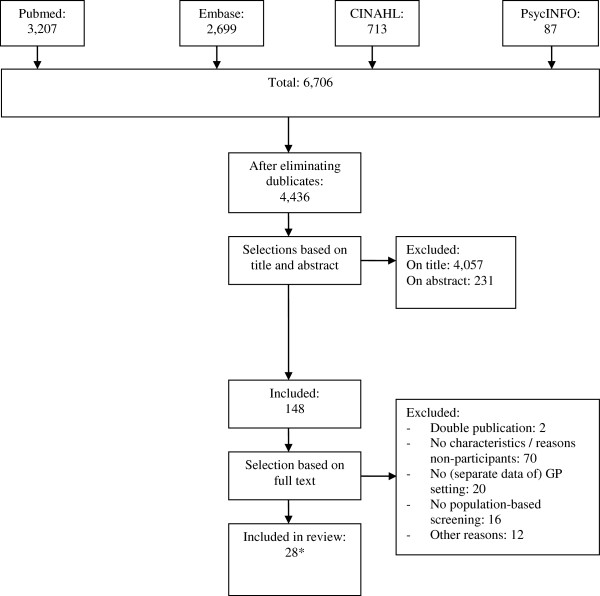
**Flow diagram outlining the study selection process.** * performed in 24 original populations.

The database search yielded 6,706 articles in total of which 4,436 remained after eliminating duplicates (see Figure 1). From the 148 articles selected on the basis of title and abstract, 30 articles were eligible for this review, based on the criteria as shown in Box 1.

Two of these articles [10,11] were excluded because of double publication of part of the results of the same large study, which were presented all together in one other article [12], leaving 28 articles for inclusion. Two of the included articles [13,14] were conducted in the same study population but with other study aims, and three studies [15-17] were performed in a subpopulation of a large study [18-20]. Therefore, this review includes studies in 24 original populations. Reasons to exclude articles after screening of the full text were lack of information about non-participation (58%), prevention program was not initiated by GPs or no separate data of the General Practice setting was presented (17%), or because the program was not a population-based prevention program (13%).

### Study characteristics

Seven of the 24 original studies described vaccination programs (five targeting influenza (described in six studies) [17,18,21-24], one Herpes zoster [25] and one Pneumococcal bacteraemia [26]), and eleven described screening programs aimed at early detection of disease (cervical cancer [27,28], Chlamydia [12,29], diabetes mellitus (described in seven studies) [9,13-16,19,20], depression [30,31], and dementia [32]). Six studies described screening programs aimed at the identification of high risk of a disease and were focused on cardio-metabolic risk [33-35], diabetes mellitus [36], fractures [37], and risk drinking [38] (Table 1). In the six studies aimed at the identification of high risk of disease, also lifestyle advice [36] treatment [37,38] or a combination of both [33,35] was offered.

**Table 1:** Characteristics and non-participation levels of included studies

**First author and year**	**Study design**	**Study size (n)**	**Country**	**Target disease**	**Inclusion criteria target group**	**Recruitment strategy**	**Non-participation level**	**Study quality#**
*Vaccination*								
Allsup [21], 2002	RCT	2583	UK	Influenza	Age (65–74), medical record	Letter + reminder	88% *	**
Arthur [22], 2002	RCT	2052	UK	Influenza	Age (≥ 75)	Letter	health check + vaccination: 26% vaccination in clinic: 32%	*
Evans [24], 2003	Observational	2600	UK	Influenza	Age (≥ 65)	Not clear	na	*
MRC Trial of Assessment and Management of Older People in the Community (MRC Study) Breeze [18], 2004 Mangtani [17], 2005 (sub study)	Observational	28492 5572	UK	Influenza	Age (≥ 75)	Letter	1997: 52% 1998: 50% 1999: 49% 2000: 37%	*
Byrnes [23], 2006	Observational	580	Australia	Influenza	Age (≥ 65)	Telephone + reminder	2004: 23% 2005: 17%	*
Opstelten [25], 2009	Observational	1778	The Netherlands	Herpes zoster and influenza	Age (≥ 65)	Letter + reminder	HZ (with and without influenza): 61%, Influenza (with and without HZ): 24%	*
Vila-Córcoles [26], 2006	Observational	10410	Spain	Pneumococcal bacteraemia/invasive disease	Age (≥ 65)	Not clear	2001: 56% 2002: 49% 2003: 48%	*
*Screening aimed at early detection of disease*								
Moser [27], 2009	Observational	3185	UK	Cervical cancer	Age (25–64), female sex	Letter	na	*
Tacken [28], 2007	Observational	2224	The Netherlands	Cervical cancer	Age (30–60), female sex	Letter + reminder	na	*
Low [12], 2005	Observational	19773	UK	Chlamydia	Age (16–39)	Letter + reminder	65%	**
Verhoeven [29], 2004	Observational	339	Belgium	Chlamydia	Age (18–35), female sex	In General Practice practice	15%	*
Pilot ADDITION trial UK: Park [13,14], 2008, 2010	RCT	355	UK	Diabetes	Age (40–69), medical record	Letter	18%	*
ADDITION trial UK: Sargeant [20], 2010 Eborall [16], 2007 (sub study)	Observational study Controlled trial	33539 7380	UK	Diabetes	Age (40–69), medical record	Letter + reminder	26%	*
ADDITION trial Denmark: Christensen [19], 2004 Dalsgaard [15], 2009 (sub study)	Observational	60926 4603	Denmark	Diabetes	Age (40–69), medical record	Letter	50%	*
Marteau [9], 2010	RCT	1272	UK	Diabetes	Age (40–69), medical record	Letter	43%	*
Van der Veen [30], 2009	Observational	8475	The Netherlands	Depression and anxiety	Age (18–65), recent contact PCP, medical record	Letter	64%	*
Yeung [31], 2006	Observational	5203	USA	Depression	Age (≥ 18), ethnicity	In General Practice practice	27%	*
Fowler [32], 2012	Observational	554	USA	Dementia	Age (≥ 65), medical record	In General Practice practice	10%	*
*Screening aimed at identification of high risk of disease*								
Vermunt [36], 2010	Observational	16032	The Netherlands	Diabetes	Age (40–70), medical record	Letter	45%	**
Van de Kerkhof [33], 2010	Observational	1704	The Netherlands	Cardio-metabolic risk	Age (40–75), medical record	Letter	25%	*
Nielen [35], 2011	Observational	9896 **	The Netherlands	Cardio-metabolic risk	Age (45–70), medical record	Letter or poster and leaflets in waiting room	Letter: 67% Poster/leaflet: 99%	**
Lambert [34], 2011	Observational	24166	UK	Cardio-metabolic risk	Age (≥ 40), male sex, medical record	Letter or telephone call	76%	*
Barr [37], 2005	Observational	5306	UK	Fractures	Age (≥ 70), female sex	Letter	32%	*
Zanjani [38], 2006	Observational	8367	USA	Risk drinking	Age (≥ 65)	Not clear	52%	*

na : not applicable.

* : low participation level mainly due to the research design; randomized controlled trial of vaccination versus placebo.

HZ: Herpes Zoster.

**: in one half of the practices, GPs invited their selected patients by mail (n = 1583) and in the other half of the practices patients were invited by posters and leaflets in the waiting room (n = 8313 patients belonging to the target group).

#: study quality. * = characteristics or reasons of non-participants given, ** = characteristics and reasons of non-participants given.

Study quality is presented in Table 1. Twenty original studies were qualified as ‘moderate’ [9,13-20,22-34,37,38]. Of these studies, one assessed reasons of non-participation [23]. Four studies were of ‘higher’ quality [12,21,35,36]. No differences were found in levels of non-participation between studies of moderate and higher quality.

### Recruitment strategies

Recruitment of the target group was performed by sending a letter on behalf of the GP in 17 studies (71%), in three studies individuals of the target group were invited in the practice prior to their consultation, regardless their complaints [29,31]. In one study the target group was partly invited by telephone [34] and in another study by using posters and leaflets in the waiting room [35]. In six studies participation levels were compared using different recruitment strategies, namely an informed choice versus a standard letter, vaccination at the clinic versus a health check plus vaccination at home, an invitation letter versus posters and leaflets in the waiting room, using telephone recruitment and booking, and loss framed versus gain framed messages (information on screening aimed at what people may lose by not participating versus what they may gain by participating) [9,14,22,23,34,35].

### Non-participation in population-based disease prevention programs

In general, non-participation levels showed a very broad range from 10% to 99% (median 38%) (see Table 1). Not only between studies targeting different diseases, but also within studies targeting the same disease, differences in non-participation levels were high.

Table 2 summarizes the significant socio-demographics and behavioural factors related to non-participation. Behavioural factors were significantly related to non-participation in all of the studies they were accounted for [13,22,24,25,28,32]. Several behavioural factors indicated an underestimation of the personal importance to participate, which was also the most cited reason of non-participation [12,21,23,31,36], followed by being unable to attend or hold on to the program [21,23,31,36], concerns about side effects [21,23], and unpleasantness of vaccination/screening [12,21] (data not shown). In addition, former non-participation was an indication of actual non-participation as well [22,24].

**Table 2:** Summary of significant socio-demographics and behavioural factors related to non-participation

**Factor (n studies used) #**	**N studies stat. sig. (%)**	**Non-participation vaccination**	**Non-participation early detection of disease**	**Non-participation identification high risk of a disease**
Age (20) [9,12-14,18-20,22,24-26,28-33,35-38]	15 (75%)	Younger [24,26] Older [18]	Younger [9,12,19,20,29,30] Youngest and oldest [28] Older [32]	Younger [33,36,38] Older [37]
Sex (17) [9,12-14,18-22,24-26,30-33,35,36,38]	9 (53%)	Female [18,21]	Male [12,19,20,30]	Female [38] Male [33,36]
SES(−related factors) * (8) [15,18,25,27,28,32,33,35]	4 (50%)	Low [18] High [25]	Low [15]	Low [33]
Behavioural factors (6) [13,22,24,25,28,32]	6 (100%)	Lack of relevance [24] Lack of net benefit [24,25] Low perceived severity [25] Barriers [25] (to much trouble, being against vaccination) Previous non-participation [22,24]	Stronger belief that physician wants women to attend screening [28] Less strong feeling a personal moral obligation [28] Higher treatment control [13] Lower negative emotional perceptions [13] Lower perceived benefit [32]	

SES(−related factors) *: Socioeconomic status (SES), education, employment, income.

# in case of use of a variable in both trial and sub study in the same population, the variable is included only once.

Less evidence was found for socio-demographic factors to play a role in non-participation. Age, sex and socioeconomic status (SES) were significantly related to non-participation in respectively 75%, 53% and 50% of the studies, but the effects of these factors remained unclear since results contradicted. However, non-participants are likely to be younger (in 11 out of 15 studies) [9,19,20,30,33,36-38] and male (in 6 out of 9 studies) [12,19,20,30,33,36]. Furthermore, there was some indication that non-participants were more likely to be living in deprived area’s [9,12,18,20] and to have a lower health consumption [9,13,20,33] (data not shown).

Health status, which was operationalized in various ways (e.g. presence of unhealthy lifestyle factors or chronic disease and self-reported health) was in both positively and negatively related to participation in an equal way. Only a few studies included physical environment and practice related factors and the effect of these factors therefore remains unclear.

More detailed information per study of factors included and factors significantly related to non-participation is presented in Additional file 3: Appendix 2.

Related to recruitment strategy, non-participation levels within comparable programs were lower in studies in which a more active recruitment strategy was used to improve uptake, including vaccination at home [22], telephone recruitment and booking [23], and recruitment in the practice compared to studies in which a personal invitation by letter was used [29,31]. Also, offering a paper [33] instead of an on-line risk questionnaire [35] resulted in higher uptake in comparable programs.

Furthermore, a more passive recruitment strategy (posters and leaflets in the waiting room) resulted in a much higher non-participation level compared to an invitation letter in the same study [35].

No clear difference could be found between the program types with respect to non-participation levels. They all showed a similar very broad range in non-participation levels (vaccination: 17% to 88% (median 32%), early detection: 10% to 65% (median 27%), and identification of high risk: 25% to 99% (median 52%)) (see Table 1). Furthermore, no indication was found of differences between the three program types with respect to factors related to non-participation.

## Discussion

To our knowledge, this is the first review on non-participation in vaccination programs as well as population-based screening programs embedded in General Practice.

In total, 28 studies in 24 original populations matched our search criteria regarding three program types: vaccination, early detection of disease, and identification of high risk of a disease. Non-participation levels ranged between 10% and 99% in all three program types. Median non-participation level was 38%. This review showed that program type does not seem to influence non-participation in population-based disease prevention programs in General Practice. In addition, no differences were found between the three program types with respect to factors related to non-participation. However, within each program type, and even within programs targeting the same disease, considerable differences were shown, which may be partly related to differences in recruitment strategies used.

Three groups were identified which might need specific attention, since they are less likely to participate in population-based disease prevention programs in General Practice; younger individuals, the people living in a socially deprived area, and former non-participants. In general, non-participants found participation not to be of personal relevance.

A previous review on vaccination programs also showed younger individuals to be less likely to participate, however, screening studies showed ambiguous outcomes of age [6,7]. Living in socially deprived area was also shown to be related to non-participation in vaccination programs [4] as well as in colorectal and cervical cancer screening [39,40]. Previous non-participation, either in the same or other programs is a strong predictor of current non-participation [6], which was also reflected in the lower likelihood of non-participation in individuals with a higher health care consumption in the diabetes screening programs [13,20]. An explanation could be that people get familiar with the tests and procedures involved and overcome their barriers.

Lack of personal relevance and low perceived risk are important factors in non-participation [7,41,42]. People may not respond since they feel and think they live healthy [4]. However, risk of disease is also a difficult concept for people to understand and often underestimated, especially in diseases that may be preventable by behavioural change [43].

The lowest non-participation levels were seen in programs applying more active and personal approaches, including recruitment in General Practice. A passive approach including recruitment by posters and leaflets in the waiting room of the General Practice practice, resulted in only little participation [35]. Personal contact with a GP or nurse therefore seems to play a decisive role in increasing participation [7]. Two other reviews showed that invitation, reminding, and counselling by telephone all seem to be effective interventions to increase uptake, however, they are much more intensive and expensive [6,44]. Furthermore, the results of this review indicate that it might be important to more specifically address to the personal relevance of vaccination and screening. Recent studies showed that the concept of risk is better understood if information is presented in terms of natural frequencies rather than as an absolute or relative risk [45]. In addition, low future time orientation, as often seen in the socially deprived, may be addressed to, since this is associated with a lower likeliness of changing (health) behaviour. Invitations should therefore stress the immediate benefits and remove barriers to e.g. participate in vaccination or screening and/or changing lifestyle [8,46].

Noticeably, relatively few programs were aimed at identification of high risk of disease. For example, only one out of eight of programs focused on diabetes was aimed at identifying high risk of this disease. The relatively low participation level in this study may have been due to the fact that the invitation letter informed individuals that when they were at high risk of diabetes they would be offered a lifestyle intervention. The prospect of having to change ones lifestyle may have discouraged some people to participate [36].

Our inclusion criteria are mainly related to health care systems in which the GP is a gatekeeper, which is the case in the Netherlands. Within this system GPs are able to pro-actively invite potentially high risk individuals within their patient population. This is reflected in the studies presented in this review. In countries with a non-gatekeeper system, the described programs would be performed in an opportunistic manner, which was an exclusion criteria in this review. Furthermore, population-based prevention programs in Europe are generally free of charge, while individuals in the USA have to pay or get reimbursement through their insurance. Generalizability of the results of this review, therefore, may be limited.

Some limitations of this systematic review can be noted. First, as with systematic reviews in general, despite of a thorough search strategy, relevant (un)published studies may have been omitted, as well as non-English or Dutch articles. Adding the concept ‘feasibility’ to the search strategy may have resulted in more included studies. Additionally, research on breast- and colon cancer screening was not retrieved since these programs need more specific equipment, which is usually not available in General Practice practices. Furthermore, although participation may have been studied, the results may not have been included in the final article. Next, the relatively small number of studies included, and the large heterogeneity in study characteristics and results require cautious interpretation of the results of this review.

Additionally, six included studies were part of the same international trial. Therefore, although we tried carefully to prevent presentation of duplicate data some bias may have been introduced. However, in the UK and Denmark arm of the study different target groups and recruitment strategies were used. In addition, the majority of the included studies were trials instead of real-world programs. This may have led to an overestimation of the participation levels, since these studies may be more likely to be performed in well-organized General Practice practices in less deprived areas. Finally, although we identified some factors that might be related to non-participation, it remains unclear from this review how these factors interrelate.

The findings of our systematic review provide guidance for future research and General Practice. Only 17% of the studies addressed both characteristics and reasons of non-participation. To gain more insight in who is not willing to participate in disease prevention programs and why, it is important to assess both.

Future research on willingness to participate should focus on programs aimed at identifying high risk of disease, since they may have great potential in improving population health but are also more demanding to people than the other two types. Additionally, research should be focused on the consequences this may have for recruitment in these programs.

Recruitment strategies should address to personal relevance, and to be tailored to the specific needs of subgroups of individuals. Those who are less likely to participate, such as individuals living in a deprived area, former non-participants and younger individuals, may need to be more actively recruited, in which contact with a health professional may be pivotal, especially when invited for the first time. For example, GPs and practice nurses may emphasize the importance of participation during regular encounters. In those who are more likely to engage in vaccination and/or screening, an invitation letter signed by the GP may be sufficient.

## Conclusion

In conclusion, in this review we did not find clear evidence of program type influencing non-participation in population-based disease prevention programs. In general, conflicting results regarding factors related to non-participation were shown, yet we did find three groups that might be less likely to participate, namely younger individuals, those living in a deprived area and former non-participants. Furthermore, higher participation levels were reached in studies with more active recruitment strategies. Therefore, there is still much to be gained by tailoring strategies to improve participation in those who are less likely to do so.

## Competing interests

The authors declare that they have no competing interests.

## Authors’ contributions

BK: conducted the literature search and review and wrote the manuscript. JCK and MMN: advised on the search strategy, were involved in the literature search and review and commented on the drafts of the manuscript. FGS: initiated and supervised the study and critically edited the manuscript. All authors read and approved the final manuscript.

## Pre-publication history

The pre-publication history for this paper can be accessed here:

http://www.biomedcentral.com/1471-2458/12/856/prepub

## Supplementary Material

Additional file 1 **Appendix 1.** Search strategy. Click here for file

Additional file 2 **Box 1.** Inclusion and exclusion criteria studies. Click here for file

Additional file 3 **Appendix 2.** Detailed information of studies of vaccination programs and screening programs aimed at early detection of disease. Click here for file
