# Supplementary material for: Non-participation in population-based disease prevention programs in general practice
Source: BMC Public Health. 2012 Oct 9;12:856. doi: 10.1186/1471-2458-12-856 (PMC3490995; doi:10.1186/1471-2458-12-856)
Supplement: Additional file 1 — Appendix 1. Search strategy. [file 1471-2458-12-856-S1.docx]

**Appendix 1: Search strategy**

| Search |  |
| --- | --- |
| #1 Prevention | "preventive medicine"[MeSH] OR "secondary prevention"[MeSH] OR "primary prevention"[MeSH] OR "secondary prevention"[TIAB] OR "primary prevention"[TIAB] OR "preventive care"[TIAB] OR "preventive therapy"[TIAB] OR "preventive treatment"[TIAB] OR "selective prevention"[TIAB] OR “programmatic prevention”[TIAB] OR “programmatic intervention”[TIAB] OR "indicated prevention"[TIAB] OR screening[TIAB] OR “mass screening”[MeSH] OR "health check"[TIAB] OR “risk check”[TIAB] OR “risk questionnaire”[TIAB] OR “risk assessment”[TIAB] OR (invitation[TIAB] AND counseling[TIAB]) OR (invitation[TIAB] AND counseling[TIAB]) OR (invitation[TIAB] AND intervention[TIAB])) NOT "tertiary prevention"[MeSH] OR "rehabilitation"[MeSH] OR "tertiary prevention"[TIAB] OR “rehabilitation”[TIAB] |
| #2 General Practice | "general practice"[MeSH] OR "family practice"[MeSH] OR "general practitioners"[MeSH] OR "primary health care"[MeSH] OR "general practice"[TIAB] OR "family practice"[TIAB] OR "general practitioners"[TIAB] OR "general practitioner"[TIAB] OR "primary health care"[TIAB] OR "primary care"[TIAB] OR "general physician"[TIAB] OR "general physicians"[TIAB] OR “family physician”[TIAB] OR “family physicians”[TIAB] |
| #3 Willingness to participate | recruitment[TIAB] OR uptake[TIAB] OR participate[TIAB] OR participation[TIAB] OR nonparticipation[TIAB] OR Response[TIAB] OR nonresponse[TIAB] OR responders[TIAB] OR “non participants”[TIAB] OR engagement[TIAB] OR compliance[TIAB] OR noncompliance[TIAB] OR adherence[TIAB] OR nonadherence[TIAB] OR Attendance[TIAB] OR nonattendance[TIAB] OR attenders[TIAB] OR retention[TIAB] OR “drop out”[TIAB] OR dropout[TIAB] OR dropouts[TIAB] OR drop-outs[TIAB] OR Acceptance[TIAB] OR nonacceptance[TIAB] |
| #4 Restrictions language and publication date | English[lang] OR Dutch[lang] AND "2000/01/01"[PDAT] : "3000"[PDAT] |
| #5 Restrictions publication type | (editorial[PT] OR letter[PT] OR comment[PT] OR interview[PT]) |
| #6 | #1 AND #2 AND #3 AND #4 NOT #5 |
