# Supplementary material for: Non-participation in population-based disease prevention programs in general practice
Source: BMC Public Health. 2012 Oct 9;12:856. doi: 10.1186/1471-2458-12-856 (PMC3490995; doi:10.1186/1471-2458-12-856)
Supplement: Additional file 2 — Box 1. Inclusion and exclusion criteria studies. [file 1471-2458-12-856-S2.docx]

**Box 1: Inclusion and exclusion criteria studies**

| **Inclusion criteria**   - the target group is specified and includes potential high risk individuals - the target group includes individuals of 18 years of age or older - the screening/vaccination initiative is with the General Practitioner - the screening/vaccination initiative is not consult-related - positive screened individuals get some kind of intervention (additional diagnostics, treatment, counselling, etc.) - the screening activity is performed in General Practice or, in case of different settings, data should be separately depicted - the study includes information on either factors related to non-participation or reasons of non-participation | **Exclusion criteria**   - prevention aimed at the individual patient instead of groups of individuals - the inclusion criteria of the target group are solely based on familial risk - the target group includes individuals with symptoms or diseases related to the target disease - the target group includes individuals under 18 years of age - qualitative studies, since they may identify why factors are important in participation, but not which - reviews, letters, editorials and comments, because they usually do not contain original studies - studies including screening of breast cancer or colon cancer, since these types of screening need more specific equipment which is usually not available in General Practice practices - studies in different settings in which results are not separately presented for General Practice |
| --- | --- |
