# Supplementary material for: Non-participation in population-based disease prevention programs in general practice
Source: BMC Public Health. 2012 Oct 9;12:856. doi: 10.1186/1471-2458-12-856 (PMC3490995; doi:10.1186/1471-2458-12-856)
Supplement: Additional file 3 — Appendix 2. Detailed information of studies of vaccination programs and screening programs aimed at early detection of disease. [file 1471-2458-12-856-S3.docx]

**Appendix 2: Detailed information of studies of vaccination programs and screening programs aimed at early detection of disease**

| **First author and year** | **Study size (n)** | **Target disease** | **Variables used to compare participants and non-participants** | **Non-participation level** | **Significant factors related to non-participation** | **Study quality#** |
| --- | --- | --- | --- | --- | --- | --- |
| *Vaccination* |  |  |  |  |  |  |
| Allsup [21], 2002 | 2583 | Influenza | Sex,  Reasons of non-participation | 88% ^ | Women | ** |
| Arthur [22], 2002 | 2052 | Influenza | Age, sex, receipt influenza vaccination in previous year, recruitment strategy: invitation to attend vaccination at clinic vs. health check + vaccination at home | health check + vaccination: 26%  vaccination in clinic: 32% | Received personalized letter instead of combined health check and vaccination  Actively declined the offer of health check: no vaccination in previous year | * |
| Evans [24], 2003 | 2600 | Influenza | Age, sex, perception of influenza illness, vaccine efficacy and safety, number of chronic illnesses, being hospital outpatient, self-reported health status, advice from doctor/nurse, sources of health advice | na | Younger age, not sharing the belief that everyone over 65 years of age should be immunized, believing that the vaccine side-effects are more risky than the disease, no previous immunization | * |
| Breeze [18], 2004  Mangtani [17], 2005 (sub study) | 28492  5572 | Influenza | Age, sex, SES, deprivation score, population density  Age, sex, SES, deprivation score, urban indicator, marital status, living status, smoking status, depression score, cognitive deficit, history of CVD, history of respiratory problems, indicator frailty, being carer for someone, social contact, having a confidant, participation in nurse assessment | 1997: 52%  1998: 50%  1999: 49%  2000: 37% | Older age, women, the most deprived, SES other than owner-occupied accommodation with central heating and those in sheltered/residential homes  Deprivation (no trend), urban indicator ≤ 2500 | * |
| Byrnes [23], 2006 | 580 | Influenza | Reasons of non-participation | 2004: 23%  2005: 17% |  | * |
| Opstelten [25], 2009 | 1778 | Herpes zoster | Age, sex, education, cues to action, perceived severity, perceived barriers,  Co-morbidity, uptake influenza vaccination | HZ (with and without influenza): 61%,  Influenza (with and without HZ): 24% | Refusing influenza and/or HZ vaccination: high education (only refusing both), not having diabetes (only refusing both), cues to action: believing that PCP does not recommend HZ vaccination, non-importance of complying to advice PCP, perceived severity (perceiving no high risk of contracting shingles, not believing pain shingles will last long), perceived barriers (believing that vaccinations weaken one’s natural defences, finding it too much trouble to go to the PCP for vaccination (only refusing both), being against vaccination in general (only refusing both)) | * |
| Vila-Córcoles [26], 2006 | 10410 | Pneumococcal bacteraemia/invasive disease | Age, sex, presence of diseases or risk factors for pneumonia | 2001: 56%  2002: 49%  2003: 48% | Younger age | * |
| *Screening aimed at early detection of disease* |  |  |  |  |  |  |
| Moser [27], 2009 | 3185 | Cervical cancer | SES, education, ethnicity, region, cars available in household, housing tenure | na | Non-White British | * |
| Tacken [28], 2007 | 2224 | Cervical cancer | Age, education, type of health insurance, urbanicity of practice location, smoking behaviour, beliefs toward screening and attendance, lifelong number of sexual partners | na | Youngest (30) and oldest (55 and 60) women, stronger belief that PCP wants woman to attend screening (normative beliefs), less strongly feeling a personal moral obligation, non or two or more lifelong numbers of sexual partners compared to one, health-authority-based approach of invitations and reminders compared to PCP-based approach or combination | * |
| Low [12], 2005 | 19773 | Chlamydia | Age, sex, ethnicity, social deprivation score practice,  reasons of non-participation | 65% | Younger age, men, patients of practices with higher deprivation scores | ** |
| Verhoeven [29], 2004 | 339 | Chlamydia | Age, risk profile | 15% | Younger age | * |
| Pilot ADDITION trial UK: Park [13,14], 2008, 2010 | 355 | Diabetes | Park, 2008: Age, sex, BMI, illness perception, anxiety, self-perceived health, prescribed cardiovascular drugs  Park, 2010: Age, sex, BMI, prescribed cardiovascular drugs, recruitment strategy: loss vs. gain frame messages | 18% | Park, 2008: Illness perception: higher treatment control, lower negative emotional perceptions.  Not on cardiovascular drugs  Park, 2010: Men: non-participation higher in gain-frame group; women: non-participation higher in loss-frame group | * |
| ADDITION trial UK:  Trial: Sargeant [20], 2010  Eborall [16], 2007 (sub study) | 33539  7380 | Diabetes | Age, sex, BMI, results previous screening steps, prescribed drugs, location and social deprivation score of practice  (State) anxiety, depression, worry about diabetes, self-reported health | 26% | Younger age, men, higher deprivation score, other than rural location, higher BMI, not on cardiovascular drugs, smaller practice size, lower prevalence of known diabetes in practice, higher PCP whole-time equivalents  Higher scores on diabetes specific worry at 3-6 and 12-15 months | * |
| ADDITION trial Denmark: Trial: Christensen [19], 2004  Dalsgaard [15], 2009 (sub study) | 60926  4603 | Diabetes | Age, sex, risk score screening    Age, sex, education, employment, income, marital status, size residence, risk score screening | 50% | Younger age, men  Younger age, low education, unemployed, low income, single | * |
| Marteau [9], 2010 | 1272 | Diabetes | Age, sex, social deprivation, BMI, prescribed drugs, recruitment strategy: informed choice vs. standard letter | 43% | Younger age, living in more deprived areas, higher BMI, not on cardiovascular drugs | * |
| Van der Veen [30], 2009 | 8475 | Depression and anxiety | Age, sex, morbidity in the last 2 years (depression, anxiety, having recorded psychological problem and/or somatic condition) | 64% | Younger age, men | * |
| Yeung [31], 2006 | 5203 | Depression | Age, sex | 27% |  | * |
| Fowler [32], 2012 | 554 | Dementia | Age, sex, education, income, ethnicity, housing and marital status, experiences with dementia, perception of dementia screening and other types of screening | 10% | Older age, lower perceived benefit of screening | * |
| *Screening aimed at identification of high risk of disease* |  |  |  |  |  |  |
| Vermunt [36], 2010 | 16032 | Diabetes | Age, sex, risk score,  reasons of non-participation | 45% | Younger age, men | ** |
| Van de Kerkhof [33], 2010 | 1704 | Cardio-metabolic risk | Age, sex, SES, ethnicity, health care utilization | 25% | Younger age, men, lower SES, non-White ethnicity, lower health care consumption | * |
| Nielen [35], 2011 | 9896^^ | Cardio-metabolic risk | Age, sex, education, ethnicity, smoking status, physical activity, BMI, alcohol use, familial history of diabetes and cardiovascular disease,  reasons of non-participation | Letter: 67%  Poster/leaflet: 99% | Higher alcohol use | ** |
| Lambert [34], 2011 | 24166 | Cardio-metabolic risk | Age, deprivation, ethnicity, smoking status, telephone number available to GP, GP payment for screening, GP type | 76% | Lowest and highest age categories, white/British, smokers, telephone number not available to GP | * |
| Barr [37], 2005 | 5306 | Fractures | Age, weight, smoking status, self-reported health, pervious falls and fractures | 32% | Older age, poorer health, higher self-reported history of falls | * |
| Zanjani [38], 2006 | 8367 | Risk drinking | Age, sex, ethnicity, marital status, alcohol and drug use, stages of change, cognitive impairment, distress/depression, support, health status | 52% | Younger age, women | * |

^: low participation level mainly due to the research design; randomized controlled trial of vaccination versus placebo

^^: in one half of the practices, GPs invited their selected patients by mail (n = 1583) and in the other half of the practices patients were invited by posters and leaflets in the waiting room (n = 8313 patients belonging to the target group)

#: study quality. * = characteristics or reasons of non-participants given, ** = characteristics and reasons of non-participants given
